# Septic Shock Resulting in Death After Operative Delivery

**DOI:** 10.1155/S1064744901000096

**Published:** 2001

**Authors:** Zehra Neşe Kavak, Alin Başgül

**Affiliations:** 1 Department of Obstetrics and Gynecology University of Marmara School of Medicine Istanbul Turkey; 2 Kısıklı Caddesi No. 140 Camlica Istanbul Turkey

## Abstract

*Background:* We report a youngwoman who developed septic shock after operative delivery in the 32nd week of pregnancy.Clinical features, treatment modalities and prognosis of this high-mortality-rate disorder are presented and discussed.

*Case:* A 24-year-old woman, gravida 1, para 1, was referred to our clinic in a confused state and immediately admitted to our emergency unit. She apparently had eclampsia antenatally. Termination of pregnancy with induction of labor and vacuum extraction had been employed in gestational week 32 of pregnancy. One day after delivery, her clinical and laboratory parameters worsened, so she was referred to our clinic. After a thorough physical examination and laboratory evaluation, the patient was diagnosed as having sepsis and disseminated intravascular coagulation. After blood and urine cultures were taken, aggressive management included volume repletion, antibiotics and positive inotropic therapy. Because she had persistent fever and unimproved laboratory values despite these therapies, the uterus and ovaries were thought to be the source of sepsis, and total abdominal hysterectomy and bilateral salpingo-oophorectomy were performed. Neither clinical nor laboratory parameters improved, and the patient died 28 days after delivery as a result of respiratory failure.

*Conclusion:* It is our purpose to emphasize that a rapid and appropriate decision for surgery may prevent the maternal mortality in obstetric septic shock patients. Successful management depends on early identification and aggressive treatment.


Infect Dis Obstet Gynecol 2001;9:51–54

### Septic shock resulting in death after operative delivery

**Zehra Neşe Kavak and Alin Başgül**

*Department of Obstetrics and Gynecology, University of Marmara School of Medicine, Istanbul, Turkey*

***Background:*** We report a young woman who developed septic shock after operative delivery in the 32nd week of pregnancy. Clinical features, treatment modalities and prognosis of this high-mortality-rate disorder are presented and discussed.

***Case:*** A 24-year-old woman, gravida 1, para 1, was referred to our clinic in a confused state and immediately admitted to our emergency unit. She apparently had eclampsia antenatally. Termination of pregnancy with induction of labor and vacuum extraction had been employed in gestational week 32 of pregnancy. One day after delivery, her clinical and laboratory parameters worsened, so she was referred to our clinic. After a thorough physical examination and laboratory evaluation, the patient was diagnosed as having sepsis and disseminated intravascular coagulation. After blood and urine cultures were taken, aggressive management included volume repletion, antibiotics and positive inotropic therapy. Because she had persistent fever and unimproved laboratory values despite these therapies, the uterus and ovaries were thought to be the source of sepsis, and total abdominal hysterectomy and bilateral salpingo-oophorectomy were performed. Neither clinical nor laboratory parameters improved, and the patient died 28 days after delivery as a result of respiratory failure.

***Conclusion:*** It is our purpose to emphasize that a rapid and appropriate decision for surgery may prevent the maternal mortality in obstetric septic shock patients. Successful management depends on early identification and aggressive treatment.

Key words: SEPSIS; DELIVERY; MANAGEMENT

Shock is one of the most difficult problems an obstetrician can face. The most frequent kind of shock seen by obstetricians is the hypovolemic type, followed by septic shock[1]. The incidence of maternal mortality related to sepsis has decreased during the past 2 decades owing to the availability of broad-spectrum antibiotics and advancements in critical care. However, genital tract sepsis remains a significant cause of maternal death, and sepsis continues to account for approximately 7.6% of maternal deaths in the United States[2,3].

We report here a young woman who developed septic shock after operative delivery in the 32nd week of pregnancy. Clinical features, treatment modalities and prognosis for this high-mortality-rate disorder are presented and discussed.

### CASE REPORT

A 24-year-old woman, gravida 1, para 1, had been referred to our clinic in a confused state and immediately admitted to the intensive care unit. She had a history of eclamptic convulsions in the antenatal period. Her pregnancy was terminated with the induction of labor and vacuum extraction in the 32nd week of pregnancy in a preferred

This case report was presented at the Congress of 7th European Conference of Infectious Diseases in Obstetrics and Gynecology, 4–6 October, 1999, Istanbul, Turkey.
Correspondence to: Zehra Neşe Kavak, Kısıklı Caddesi No. 140, Camlica, Istanbul, Turkey.

hospital. One day after the delivery, her clinical and laboratory parameters worsened, so she was referred to our hospital in the second postpartum day. As a result of a thorough physical examination and laboratory evaluation, it was found that she had a fever of 39.5°C, moderate tachypnea, and moderate dyspnea. On the same day of her admittance to the intensive care unit, she was intubated. She had also severe anemia, prolonged prothrombin time and elevated liver enzymes, elevated lactic dehydrogenase values, elevated blood urea nitrogen and creatinine values, elevated fibrin degradation products, increase in D-dimer fraction, low white blood cell count and very low platelet count. Her arterial blood gas measurement showed hypoxemia and metabolic acidosis. Her abdominal ultrasound showed hepatosplenomegaly and ascites, and her chest radiograph showed bilateral infiltration of the lungs. In addition, repeated pelvic ultrasound revealed a very thick endometrial lining. Revisional uterine curettage was performed in the intensive care unit on the following day to evacuate infected products from the uterus and to take endometrial culture. The patient was diagnosed as having sepsis, resulting in disseminated intravascular coagulation and acute renal failure.

Hemodialysis was started according to her needs. Blood and urine cultures were taken, and aggressive management was undertaken with oxygen support with variable intensity, volume repletion which was fresh frozen plasma, thrombocyte suspension, erythrocyte suspension 5% dextrose in water, and 0.9% isotonic and lactated Ringer's solution. In an attempt to maintain the physiologic milieu, minimizing the severity of organ failure, plasmapheresis was begun. The nephrology department of our hospital made this decision to eliminate high-molecular-weight cytokines. Plasmapheresis was performed five times over the following 5 days.

Many different types of broad-spectrum antibiotics in increasing dosages and in different combinations were used, which included menopenem, teicoplanin, sulbactam plus cefoperazone, trimetoprim plus sulfamethoxozole, ceftazidime pentahydrate, flucanozole and amikacin. Antibiotics were changed according to the patient's response to the treatment and blood culture results by the intensive care unit team. Positive inotropic therapy with dopamine and, later, according to the needs of the patient, additional dobutamine and epinephrine infusion with increasing dosages also were given.

The fulminant septic process could not be stopped with conservative treatment. She had a persistent high-grade fever and unimproved laboratory values despite therapies given over 10 days. We decided to operate on the patient. During the operation, 4 liters of infected, green, malodorous ascites were aspirated. Total abdominal hysterectomy and bilateral salpingo-oophorectomy were performed.

On exploration, nearly a 4 × 5 cm pancreatic mass was palpated, and a pancreatic biopsy was taken. A pathologic report on the specimens detailed a hemorrhagic infarction in the endometrium and acute pancreatitis. Repeated blood cultures on different days revealed *Acinetobacter, Enterococcus, Pseudomonas* and *Candida,* and endometrial cultures showed *Staphylococcus aureus*. Despite the intensive care unit support and aggressive treatment, neither clinic nor laboratory parameters improved, and the patient died 28 days after delivery as a result of respiratory failure.

### DISCUSSION

Sepsis, the inflammatory response to an infection, is a leading cause of death in intensive care units[4]. Mortality rates range from 20% to 90% despite our growing understanding of the pathogenesis of sepsis and advances in therapy. Every year in the United States, there are an estimated 400 000 cases of sepsis; half of these are severe enough to result in shock and are responsible for more than 100 000 deaths per year[4,5].

The cases of nonhemorrhagic obstetric shock (pulmonary thromboembolism, amniotic fluid embolism, acute uterine inversion and sepsis) are uncommon but are responsible for the majority of maternal deaths in the developed world[3]. Human septic shock is characterized by the presence of cardiovascular instability, severe hypotension and development of organ dysfunction[6].

Two or more of the following conditions as a result of the infection are the usual findings: temperature > 38°C or < 36°C, heart rate > 90 beats/min, respiratory rate > 20 breaths/min or arterial carbon dioxide pressure < 32 mmHg, white blood cell count > 12 000/μl, < 4000/μl or > 10% immature (band) forms[7]. Our patient had nearly all of these conditions. Because she showed so many signs and symptoms of sepsis, the diagnosis was predictable on her admittance to our hospital, even without urine and blood culture results. Although septic shock in obstetrics is most commonly associated with infection caused by endotoxin-releasing aerobic coliform organisms, it may also result from infection with other bacteria, fungi, protozoa or viruses[4]. Towards her last days, blood cultures showed superimposed fungal infection.

The earliest symptom resulting from septic shock may be an altered mental status, which is usually associated with temperature instability from the effect of endotoxin on hypothalamic temperature regulation. The most common cause of death in patients with this condition is respiratory insufficiency secondary to adult respiratory distress syndrome[7].

Both sepsis and particularly septic shock are important risk factors for the development of acute renal failure[8]. The most serious forms of acute renal failure are found in patients in intensive care units[9]. The decision to initiate renal replacement therapy is usually based on a careful assessment of conflicting priorities in the care of critically ill patients. There are no scientifically established criteria for the initiation of renal replacement therapy in critically ill patients[10]. After hemodialysis we also used plasmapheresis in our patient with the hope of removing potentially pathogenic macromolecules. Koepp and Lampert described use of plasmapheresis in a 62-year-old patient with a severe infection, and they had a temporary improvement of their patient during plasmapheresis[11]. The authors hypothesized that elimination of high-molecular-weight cytokines and toxins contributed to the improvement under plasmapheresis. When using plasmapheresis, one has to consider the high costs, the risk of infection and the unexplained mode of action on the mediator process[11]. Our patient did not respond to this treatment.

When sepsis is recognized and treated in its early stages, survival and morbidity are significantly improved. Therefore, caring for the patient in shock, although challenging, may well be rewarding[7]. Successful management of obstetric shock depends on early identification and aggressive treatment focused on stabilization of the patient, removal of underlying causes of sepsis, broad-spectrum antibiotic coverage and treatment of associated complications[4,7]. Laparatomy with possible hysterectomy should be performed if there is failure to respond to uterine evacuation and medical therapy, uterine perforation with necrotic myometrium or suspected bowel injury[12].

In fact, we tried all of the above therapeutic modalities for our patient. However, there may have been faults in the management of the patient. First, we think that, had she been diagnosed and treated before she was referred to our hospital, she might have had a better chance for survival. Second, had we operated on her within 72 h of our conservative treatment, instead of waiting for her response to changing treatment modalities for 10 days, we believe that she may have had a chance for survival.

Rivlin and Hunt found significant differences in mortality rates of puerperal sepsis. The mortality rate was lower with surgery within 24 h rather than after 24 h in 176 patients[13]. This series emphasizes the overriding importance of early surgery. However Saver and colleagues reported five cases of puerperal sepsis[14]. After 12–72 h of conservative treatment, because of clinical deterioration, all women underwent laporatomy, with hysterectomy in three cases. Although the inflammatory 'septic' source was removed by the surgical intervention, the clinical condition of three of the patients deteriorated further[14].

Nathan and co-workers presented two cases of puerperal sepsis caused by group A streptococcus in 1993[15]. Although one of the patients, who underwent hysterectomy on the 12th day of delivery died, the other patient who was treated surgically on the 5th postpartum day survived[15].

In our patient, one of our findings was a pancreatic mass, which was discovered during the operation. It was proved to be acute pancreatitis histologically. Acute pancreatitis and pregnancy are a rare and serious combination that pose difficult diagnostic and therapeutic problems[16]. The diagnosis of pancreatitis may be more difficult to confirm; some normal pregnancies associated with increased serum amylase concentration and mild to moderate elevation in many parameters are associated with peritoneal irritation and with certain medical problems as well[17].

In our patient we did not discover pancreatitis until the operation. Her amylase values were moderately increased. We do not know whether this phenomenon was present before the labor or how much it participated in the occurrence or the irreversibility of septic shock.

### CONCLUSIONS

It is our purpose to emphasize that a rapid and opportune decision for surgery may prevent patient deterioration and maternal mortality, and successful management depends on early identification and aggressive treatment.

### REFERENCES

1. Bonfante RE, Ahued AR, Garcia BCO, *et al.* Shock in obstetrics. Institutional experience. *Gynecol Obstet Mex* 1997;65:137–40
2. Lee W, Clark SW, Cotton DB, *et al.* Septic shock during pregnancy. *Am J Obstet Gynecol* 1988; 158:6
3. Thomson AJ, Greer IA. Non-hemorrhagic obstetric shock. *Baillieres Best Pract Res Clin Obstet Gynecol* 2000;14:19–41
4. Hines DW, Lisowski JM, Bore RC. Sepsis. In Gorbach SL, Barlett JG, Blocklow NR, eds. *Infectious Diseases*, 2nd edn. Philadelphia: W.B. Saunders Company, 1998:654–62
5. Parillo JE. Septic shock in humans: advances in the understanding of pathogenesis, cardiovascular dysfunction and therapy. *Ann Intern Med* 1990;113:227–44
6. Bellomo R, Boldwin I, Cole L, Ronco C. Preliminary experience with high-volume hemofiltration in human septic shock. *Kidney Int* 1998; 53:182–5
7. Smith HO. Shock in the gynecologic patient. In Rock JA, Thompson JD, eds. *Linde's Operative Gynecology*, 8th edn. Philadelphia: 1997:245–62
8. Thijs A,Thijs LG. Pathogenesis of renal failure in sepsis. *Kidney Int* 1998;53:34–7
9. Lameire N, Biesen WV. Vanholder R, Colardijn F. The place of intermittent hemodialysis in the treatment of acute renal failure in the ICU patient. *Kidney Int* 1998;53:110–19
10. Bellomo R, Ronco C. Indications and criteria for initiating renal replacement therapy in the intensive care unit. *Kidney Int* 1998;53:106–9
11. Koepp A, Lampert R. Use of plasmapheresis in a 62-year-old patient with severe infection. *Infusionsther Transfusionsmed* 1996;23:92–6
12. Hoyme UB, Kician N, Eschenbach DA. The microbiology and treatment of late postpartum endometritis. *Obstet Gynecol* 1986;68:226–32
13. Rivlin ME, Hunt JA. Surgical management of diffuse peritonitis complicating obstetric/ gynecologic infections. *Obstet Gynecol* 1986;67: 652–6
14. Saver I, Schröder W, Raumans J, Fath W. Sepsis and SIRS in the puerperium – pathogenesis and clinical management. *Z Geburtschilfe Neonat* 1998; 202: 30–4
15. Nathan L, Peters UT, Ahmed AM, *et al. Am J Obstet Gynecol* 1993;169:571–2
16. El Mansari O, Zenter A, Mewdare A, *et al.* Acute pancreatitis in the postpartum period. Apropos of 3 cases. *J Chir* 1996;133:127–31
17. Bartelink AK, Gimbrere JS, Schoots F, Dony JM. Maternal survival after acute haemorrhagic pancreatitis complicating late pregnancy. *Eur J Obstet Gynecol Reprod Biol* 1988;29:41–50

RECEIVED 7/25/00; ACCEPTED 11/28/00
